# Electronic patient-reported adverse event monitoring in academic early-phase clinical trials: A feasibility study

**DOI:** 10.1177/17407745251378668

**Published:** 2025-10-27

**Authors:** Leanne Shearsmith, Sarah Danson, Sarah Gelcich, Andrea Gibson, Kathryn Gordon, Fiona Collinson, Julie Croft, Emma Griffiths, Zoe Rogers, Robert Carter, Julia Brown, Galina Velikova, Fiona Kennedy

**Affiliations:** 1 Patient Reported Outcomes Group, Section of Patient-Centred Outcomes Research, Leeds Institute of Medical Research (LIMR) at St James’s, University of Leeds, Leeds, UK; 2 Weston Park Cancer Centre, University of Sheffield & Sheffield Teaching Hospitals NHS Foundation Trust, Sheffield, UK; 3 Section of Cancer Biology and Therapeutics, School of Medicine and Population Health, University of Sheffield, Sheffield, UK; 4 Leeds Teaching Hospitals NHS Trust, Leeds, UK; 5 Clinical Trial Research Unit, Leeds Institute of Clinical Trials Research, University of Leeds, Leeds, UK

**Keywords:** Adverse events, symptoms, oncology, electronic patient-reported outcomes, early-phase clinical trial, compliance

## Abstract

**Background:**

Adverse event monitoring is essential to monitor safety for oncology patients on early-phase clinical trials. Previous research considers that electronic patient-reported adverse events reporting is feasible and complementary to traditional clinician-led recording. An electronic patient-reported adverse event system was developed to explore the feasibility of this in early trials patients.

**Methods:**

A prospective single-arm feasibility study was undertaken at two recruiting hospitals. Participants were adult oncology patients who had recently (<1 month) started receiving a novel anticancer treatment on an academic early-phase trial and had access to the Internet. For a 12-week period, weekly reminders were sent to participants to complete an electronic patient-reported adverse event questionnaire remotely covering symptoms identified as relevant to the recruiting trials. The primary outcome was compliance (proportion of completed questionnaires/expected completions). Secondary outcomes included recruitment rates, attrition, electronic patient-reported outcome versus clinician-recorded adverse events, number of notifications, issues recorded, and patient acceptability.

**Results:**

Twenty-three participants consented (76.7% consent rate), 18 remained on study at 12 weeks (4 were withdrawn due to toxicity and 1 patient choice). Compliance with weekly electronic patient-reported adverse event was high, with a cumulative of 85.1% across the 12 weeks. Comparison with clinician-recorded adverse events showed electronic patient-reported adverse event resulted in wider coverage of adverse events: three times as many symptoms reported on electronic patient-reported adverse event (n = 174 last assessment) than recorded in the medical charts (n = 50 last record). End-of-study feedback indicated most patients reflected positively on their time on the study.

**Conclusions:**

Remote electronic patient-reported adverse event reporting by patients in early-phase trials is feasible and acceptable. The study highlights some logistical challenges that require consideration in future electronic patient-reported outcome work to ensure adverse events are fully captured and recorded.

**Trial registration:**

ClinicalTrials.gov ID: NCT03461939 (first registered: 05/03/2018)

## Introduction

The value of collecting patient-reported outcomes (PRO), which are unfiltered by clinicians has been recognised and implemented in Phase III clinical trials, usually as a secondary outcome.^1–3^ PROs, including health-related quality-of-life (HRQOL) provide important information on patient symptom or adverse events experiences and their impact on daily lives. Symptoms and adverse event (AE) focused PROs have been recommended for the evaluation of treatment tolerability of long-term oral anticancer drugs.^4^

Basch and colleagues recently highlighted the need for new drug development to add a focus on patient experiences through the use of patient-derived AE data.^5^ However, PROs are still not routinely used in early-phase trials.^6,7^ The collection of detailed AEs data in those trials is of paramount importance to detect any new and dose-limiting toxicities, but the standard protocols rely entirely on clinician reported AEs (CTCAE),^8^ despite the knowledge that clinicians downgrade the severity of symptoms and under-report lower grade or subjective symptoms (e.g. fatigue).^9,10^

Early-phase trials pose unique challenges for PRO data collection and interpretation. Participants are often frail due to advanced cancer, with multiple symptoms making it difficult to distinguish disease-related symptoms from drug-related toxicity.^11^ Furthermore, the adverse effects of conceptually new treatments are not known, so the traditional static PRO questionnaires are unlikely to capture unexpected AEs. Typically, the early-phase trials have a smaller sample (tens rather than hundreds of patients) thus raising concern about the wider validity of the PROs. An international survey of stakeholders (trialists, including clinicians, statisticians, trial managers, regulators) reported minimal use of PROs in dose-funding oncology trials, but supported their use.^12^

However, the wider use of electronic PROs (ePROs), with the increased ease of administration and real-time data collection would enable data to be collected remotely and frequently,^13^ identify new toxicities and provide additional information on frequency and duration^14^ but it may generate new ethical and logistical challenges.^15,16^ ePRO methods are implemented in early trial settings outside oncology.^17^

Our previous qualitative work explored the views of ePRO for AE (ePRO-AE) reporting among oncology early trials patients, clinicians and trial-related staff. The majority saw the benefit of ePRO-AE reporting for more comprehensive and accurate toxicity records.^18^ Concerns raised centred on the PRO-AE data flows (e.g. direct to the clinical teams or direct to the trial office) and a potential increase in clinical workload.

This small feasibility study collected weekly online ePRO-AE reports from patients registered on phase I/II oncology trials. The primary outcome was patient compliance, with secondary outcomes of recruitment rates, attrition, patient acceptability, the number of alerts for serious AEs generated by the system and recording any issues encountered.

## Method

### Design and participants

A prospective single-arm feasibility study recruited a convenience sample of patients enrolled in phase I/II early-phase ongoing academic trials from two UK hospitals between August 2018 and April 2021. Commercial trials were excluded due to the complexity of regulatory approvals. Ethical approval was granted from the Health Research Authority (HRA) National Health Service (NHS) Research Ethics Committee (18/YH/0204).

Patients were eligible if they were within 1 month of starting systemic anticancer trial treatment (e.g. chemotherapy, targeted agents, chemo-radiotherapy or chemo-immunotherapy), expected to continue the trial for at least 3 months, spoke/understood English, had access to the Internet and were aged 18 years or over. Patients with overt psychopathology/serious cognitive dysfunction were excluded.

### The electronic system and questionnaires

The secure online web-based platform (called QTool) was originally developed for cancer survivors^19^ and patients on anticancer treatments.^20,21^ Participants access the platform from home computers or mobile devices. A feasibility study found high acceptability and reporting compliance (75%–80%) for collecting PRO-AEs and quality-of-life data from patients on various treatments.^22^

The AE items for the ePRO system were selected from the National Cancer Institute (NCI) PRO-CTCAE 126-item bank, based on existing recommendations.^3,14^ The list included common symptoms for any early-phase trials (fatigue, insomnia, pain, anorexia, dyspnoea, cognitive problems, anxiety, nausea, depression/sadness, neuropathy, constipation and diarrhoea), plus trial-specific symptoms based on the protocols (e.g. flu-like symptoms, eye symptoms) **(Supplement S1).** The NCI PRO-CTCAE item bank contains 1–3 items per symptom rated for frequency, severity, and interference with everyday life.^23^ Five free-text boxes collected ‘other’ symptoms, and if reported they were rated for frequency/severity. Participants were prompted to contact their clinical team immediately, if they reported severe symptoms requiring medical attention. The system generated alerts to the study/clinical team. For any moderate or mild symptoms, the participant advice was to raise these issues on their next trial visit, or sooner, if getting worse.

### Procedure

Patients were approached by their oncology team with the information sheet. Researchers contacted interested patients to discuss the study and answer any questions. Written informed consent was obtained from all participants. Participants were invited to complete ePRO-AE items for up to 12 weeks during the early-phase trial participation with weekly reminders (email/text), but the ePRO-AE questionnaire was available for reporting at any time. They were given a step-by-step ePRO user-manual and were reminded (verbally and written, in the information sheet and the online questionnaire) that the ePRO system was not intended to replace the usual care/methods of reporting any AEs to their clinical care teams.

Demographic details and computer use information were self-reported at baseline. At the end of the study (withdrawal or 12 weeks), participants were invited to provide feedback in a telephone interview or questionnaire.

### Sample, study outcomes and analysis

The primary outcome of compliance was defined as the proportion of completed questionnaires out of expected weekly completions (excluding multiple completions per week). The number of completions per participant was calculated, including multiple completions. Secondary outcomes included recruitment rates, attrition (withdrawals), time to complete, ePRO/clinician-recorded AE comparisons, number of alerts, issues encountered and patient acceptability/feedback. Comparison between ePRO and clinician-recorded toxicity assessments (as recorded in the medical charts) was performed at baseline (first) ePRO entry and the last completion. Where ePRO symptoms were not mentioned in medical charts, the ePRO data was descriptively explored to see which symptoms were missed **(Supplement S2).**

Patient feedback was described qualitatively (thematic analysis) from the end-of-study interviews and quantitatively (end-of-study survey). Data analysis was undertaken using SPSS (version 27).

## Results

**Figure 1.**
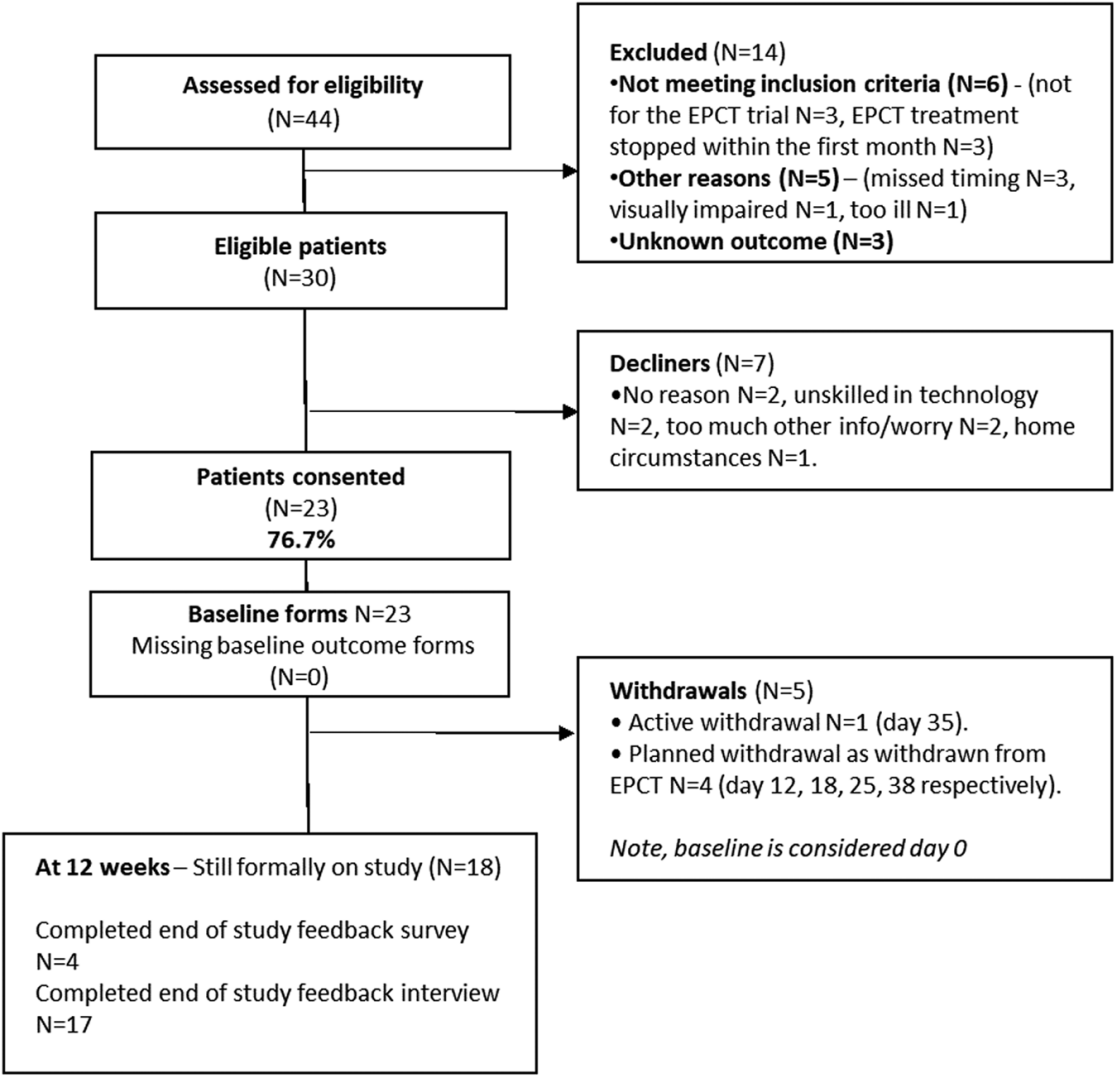
CONSORT diagram. The study recruitment was suspended for 6+ months during the coronavirus pandemic (March to September 2020, reopened September/November 2020).

From 44 patients evaluated for eligibility, 14 were not eligible (not starting/continuing the trial or missed time window for approach); 7 of 30 approached declined (2-not using computers), therefore, the consent rate was 76.7%; 23/30 eligible patients (Figure 1). At week 12, 78.3% participants (18/23) remained on study, with 1 active withdrawal at week 5 (4.3%). The patient was concerned that the ePRO-AE report may influence a potential treatment withdrawal.

Table 1 shows the demographic and clinical characteristics of the sample. All participants were enrolled in phase II trials, with equal split male/female participants, 65 years median age and a range of cancer sites. **(Supplement S3).**

**Table 1.** Demographic and clinical characteristics.

	Sample, n = 23	
**Age**		
Mean (standard deviation, SD)	64.4 (9.14)	
Median	65.0	
Min, max	40.0, 77.0	
**Time since early-phase trial start (days)**		
Mean (standard deviation, SD)	12.2 (10.63)	
Median	13.0	
Min, max	0.0, 36.0	
**Time since diagnosis (months)**		
Mean (standard deviation, SD)	37.1 (71.30)	
Median	2.0	
Min, max	0.0, 265.0	
**Site**	**n**	**%**
Site 1	13	56.5
Site 2	10	43.5
**Gender**		
Male	13	56.5
Female	10	43.5
**Marital status**		
Married/civil partnership	17	73.9
Co-habiting	2	8.7
Separated/divorced	0	0
Widowed	3	13.0
Single	1	4.3
**Education**		
Basic school	7	30.4
Education beyond minimum school leaving age	10	43.5
Higher degree or professional qualification	6	26.1
How often use a computer? *		
Daily	20	87.0
Weekly	2	8.7
Monthly or rarely	0	0
**Cancer site**		
Bladder	5	21.7
Breast	5	21.7
Renal	8	34.8
Leukaemia	1	4.3
Lung	1	4.3
Myeloma	1	4.3
Oesophageal	2	8.7
**Tumour stage**		
Locally advanced	6	26.1
Metastatic	15	65.2
Not available†	2	8.7
**Current ECOG performance status**		
0	12	52.2
1	11	47.8
**EPCT details**		
Phase I	0	0
Phase II	23	100.0
•Modified arm	11	47.8
•Standard arm	7	30.4
•Trial with various arms	4	17.4
•Double blind trial	1	4.3
**Current trial treatments**		
Systemic treatments	23	100.0
•*Chemotherapy*	13	56.5
•*Targeted agent* §	6	26.1
•*Immunotherapy*	9	39.1
•*DNA methyltransferase inhibitors*	2	8.7
Radiotherapy	5	21.7
**Comorbidities**		
None	12	52.2
1 condition	9	39.1
2 conditions	2	8.7

* There was one missing response for this question.

† No tumour stage was recorded by the two haematological patients.

§ One patient was on a double-blinded trial of a targeted agent so could have been receiving it or a placebo.

Completion rates are presented in Table 2. There were 202 ePRO completions during the entire study period (n = 22 baseline, n = 172 weekly completions (weeks 1–12) and 8 ‘additional’ i.e. 2 per week). The overall compliance rate was 85.1% (172/202 expected timepoints), with timepoint weekly compliance ≥ 75%. Nine participants completed 1–7 responses (5 of these withdrew/were withdrawn), 13 completed 9–13 responses. At the participant level, 11/23 participants had no missing completions (47.8%), whereas 7/23 participants missed one completion (30.4%), and 5/23 (21.7%) missed 3–6.

**Table 2.** Time point level data for actual/expected completions and compliance %.

Timepoint	Actual	Expected	Compliance %	Missing, when expected	Missing, when not expected*
*Week 1*	18	22	81.8	4	1 (W)
*Week 2*	17	19	89.5	2	4 (1 W, 3 IT)
*Week 3*	16	16	100.0	0	7 (3 W, 4 IT)
*Week 4*	13	16	81.3	3	7 (3 W, 4 IT)
*Week 5*	13	15	86.7	2	8 (4 W, 4 IT)
*Week 6*	12	14	85.7	2	9 (5 W, 4 IT)
*Week 7*	14	15	93.3	1	8 (5 W, 3 IT)
*Week 8*	15	17	88.2	2	6 (5 W, 1 IT)
*Week 9*	15	18	83.3	3	5 (W)
*Week 10*	15	18	83.3	3	5 (W)
*Week 11*	12	16	75.0	4	7 (5 W, 2 IT)
*Week 12*	12	16	75.0	4	7 (5 W, 2 IT)
**Overall compliance over 12**-week **period**	**172** ^#^	**202**	**85.1**	**30**	**74 (47** **W, 27 IT)**

* Reasons for not expected completions include the patient being withdrawn (W) and a problem with IT access issue (IT). The IT problems affected access to the ePRO website for six participants, including the participant that did not complete any post-baseline and three of the patients who completed less than eight responses.

# Excludes N = 8 completions which were classed as additional completions (i.e. two completions received in a 7-day window), and excluded from compliance results.

The mean time for ePROs completion was 7.5 min (standard deviation 6.9, median 6 minutes, range 1:52–59:04).

**Figure 2.**
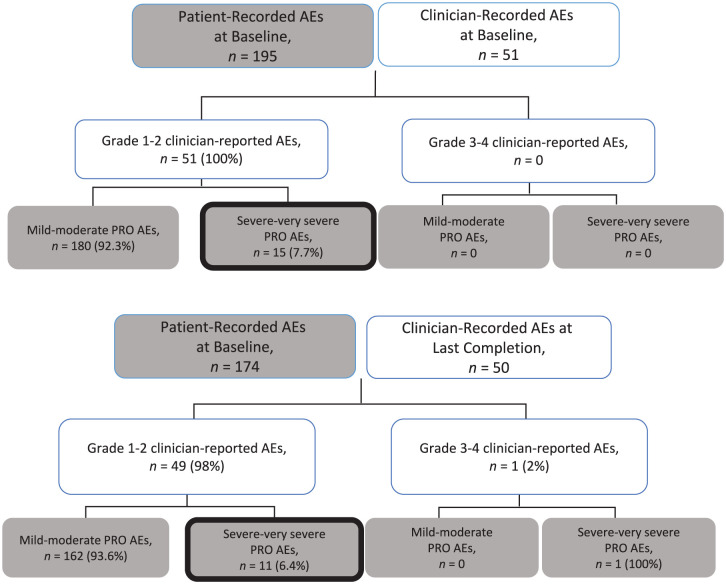
Clinician-recorded AEs (white boxes) and ePRO-AEs patient scores (grey boxes) reported on the baseline completion and the last completion time point (n=23). Note, discordant scores are highlighted with a thick black border.

The ePROs provided three times as many symptoms (n = 195 baseline; n = 174 last) than recorded by clinicians in the medical charts (n = 51 baseline; n = 50 last) (Figure 2). Among the ePRO data 7% of symptoms (N = 26/369) were rated as severe, whereas 99.0% (100/101) of clinician-recorded symptoms were low grade 1 or 2. The symptoms with agreement at baseline were fatigue (n = 14/23, 60.9%), pain (n = 10/23, 43.5%), diarrhoea (n = 10/23, 43.5%), vomiting (n = 9/23, 39.1%), nausea (n = 8/23, 34.8%), mucositis (n = 8/23, 34.8%). The overall rate of agreement across all symptoms was low at 18.2%. A similar pattern was seen at the last completion **(Supplement S4).**

Depression, insomnia, anxiety, decreased appetite and dizziness were the symptoms reported as ePROs but not recorded in the medical charts **(Supplement S5).** Patients rated them as mild (65.7%, 151/230), moderate (30.4%, 70/230), severe/frequent (2.6%, 6/230), and very severe/almost constant (1.3%, 3/230).

Eighty-one individual symptom alerts were received across all participants during the study period, related to 46 questionnaire completions (22.8%, 46/202) (**Supplementary S6**). Fifteen out of 20 individual symptoms generated alerts (most common fatigue (n = 17), and pain (n = 12)). ‘Other’ severe symptoms were reported on 14 occasions.

Patient feedback (4 end-of-study surveys, 17 interviews, duration 10–54 min) was positive, ePROs completions increased symptom awareness, symptoms were relevant, survey length and weekly completions were not a burden. Some participants preferred direct clinical contact as already seen regularly (**Supplement S7**).

## Discussion

This study represents one of the first attempts of using an ePRO system with patients enrolled in early-phase oncology trials, allowing them to record and report AEs online. The results indicate patient willingness to take part (76.7% consent rate), low attrition and good patient compliance with the weekly AE reporting (85.1% of all expected completions, > 75% at each timepoint). Compared to previous research, this study had a similar level of high compliance,^13,22^ especially given that the patients completed the ePRO reports remotely, out of hospital rather than at clinical encounters/outpatient appointments like in the previous phase I research.^7,24^ Consistent with other studies,^24,25^ the patient feedback did not indicate the reporting was burdensome, despite this being a concern raised by clinicians.^18^ Similarly, the secondary outcomes of recruitments rate, attrition and general patient acceptability were positive.

The contrast between the large number of symptoms reported by patients and the number recorded in trial/medical records shows how valuable the ePRO data can be in highlighting a full picture of patients symptoms/AEs. This discrepancy has been illustrated in previous research,^26,27^ with several possible explanations. Symptoms/AEs that patients were not experiencing were generally not recorded in trial/medical records, whereas the ePROs captured all symptoms each week. It is also possible that patients refrain from verbally communicating all their symptoms to their clinicians for fear of being taken off the trial drug. This was certainly a concern in qualitative work with early trial patients,^18^ and was the reason one participant withdrew from the current study.

Overall, in 22.8% (46/202) of completions, an email alert indicating severe/high frequency symptoms was generated, which may raise concerns about an increased workload and patient safety. Clinicians highlighted that for ePRO-AEs data to be used in safety monitoring, trials capacity would require specific standard operating procedures and out-of-hours pathways.

Limitations of the study include the small sample size from two centres and challenges of performing the study across several trials with different data collection processes. Although the consent rate among eligible patients was > 75%, 21/44 (47.7%) of the evaluated population did not participate (14 ineligible patients and 7 declined), raising concerns about the wider applicability of the study results. The main reasons for ineligibility were not continuing the trial or administrative issues (patients missed). Of note, patients were approached separately for the ePRO reporting study, after consenting to the main trial. If ePROs are planned as part of the early-phase trials, similar to the design of phase III trials, then the above issues could be avoided.

We were restricted to the academic oncology trials that were open (all phase II trials) at the time of recruitment, due to the complexities of accessing commercially funded trials (note, phase I trials are often commercial). Therefore, our conclusions may only apply to non-commercial phase II trial patients, although similar findings have been reported in some phase I studies.^7,11^ This study’s recruitment was disrupted due to the COVID-19 pandemic, where many NHS Trusts had to halt recruitment to trials.

## Conclusion

This study provides early evidence that patients enrolled in early-phase oncology trials are able to report ePRO-AE regularly, independently and using electronic methods. The patient’s voice unfiltered by a clinician may enable the full extent of the symptoms and AEs to be captured in trial records.

## Supplemental Material

sj-docx-1-ctj-10.1177_17407745251378668 – Supplemental material for Electronic patient-reported adverse event monitoring in academic early-phase clinical trials: A feasibility study Supplemental material, sj-docx-1-ctj-10.1177_17407745251378668 for Electronic patient-reported adverse event monitoring in academic early-phase clinical trials: A feasibility study by Leanne Shearsmith, Sarah Danson, Sarah Gelcich, Andrea Gibson, Kathryn Gordon, Fiona Collinson, Julie Croft, Emma Griffiths, Zoe Rogers, Robert Carter, Julia Brown, Galina Velikova and Fiona Kennedy in Clinical Trials
