# Supplementary material for: Electronic patient-reported adverse event monitoring in academic early-phase clinical trials: A feasibility study
Source: Clin Trials. 2025 Oct 27;23(1):113–20. doi: 10.1177/17407745251378668 (PMC12909613; doi:10.1177/17407745251378668)
Supplement: sj-docx-1-ctj-10.1177_17407745251378668 – Supplemental material for Electronic patient-reported adverse event monitoring in academic early-phase clinical trials: A feasibility study [file sj-docx-1-ctj-10.1177_17407745251378668.docx]

**Supplementary File**

**Content**

| Section | Page |
| --- | --- |
| S1. e PRO-AE symptom items presented to participants | 2 |
| S2. Method of comparison between clinician-recorded AEs in trial/medical records and PRO-AEs | 7 |
| S3. Early phase trial participation amongst the ePRIME participants | 8 |
| S4. Symptom level comparison between patient and clinician records at baseline and last completion timepoint | 9 |
| S5. Exploration of ‘not mentioned’ clinician symptoms and ePRO scoring at baseline and last completion timepoint | 11 |
| S6. Counts of symptoms generating notification email alerts | 13 |
| S7. Summary of patient feedback and other issues recording during the study | 14 |

**S1.** **ePRO-AE symptom items presented to participants**

|  | **Symptom items included in PRO AE system questionnaire** | **Response options for alert notifications (note ^#^ for generic responses)** | **Notification alert?** | **Dependencies** |
| --- | --- | --- | --- | --- |
| **1*** | In the last 7 days, how OFTEN did you have **pain**? |  | No | None |
| **1a** | In the last 7 days, what was the SEVERITY of your **pain** at its WORST? | None |  | 1 |
|  |  | Mild | No |  |
|  |  | Moderate | No |  |
|  |  | Severe | Yes |  |
|  |  | Very severe | Yes |  |
| **1b** | In the last 7 days, how much did **pain** INTERFERE with your usual or daily activities? |  | No | 1 |
| **2*** | In the last 7 days, how OFTEN did you have **nausea**? |  | No | None |
| **2a** | In the last 7 days, what was the SEVERITY of your **nausea** at its WORST? | None |  | 2 |
|  |  | Mild | No |  |
|  |  | Moderate | No |  |
|  |  | Severe | Yes |  |
|  |  | Very severe | Yes |  |
| **3** | In the last 7 days, how OFTEN did you have **vomiting**? |  | No | None |
| **3a** | In the last 7 days, what was the SEVERITY of your **vomiting** at its WORST? | None |  | 3 |
|  |  | Mild | No |  |
|  |  | Moderate | No |  |
|  |  | Severe | Yes |  |
|  |  | Very severe | Yes |  |
| **4*** | In the last 7 days, what was the SEVERITY of your **decreased** **appetite** at its WORST: | None |  | None |
|  |  | Mild | No |  |
|  |  | Moderate | No |  |
|  |  | Severe | Yes |  |
|  |  | Very severe | Yes |  |
| **4a** | In the last 7 days, how much did **decreased appetite** INTERFERE with your usual or daily activities: |  | No | 4 |
| **5*** | In the last 7 days, how OFTEN did you have **loose or watery stools** (diarrhoea)? | Never |  | None |
|  |  | Rarely | No |  |
|  |  | Occasionally | No |  |
|  |  | Frequently | Yes |  |
|  |  | Almost constantly | Yes |  |
| **6*** | In the last 7 days, how OFTEN did you **lose control of bowel** **movements**? | Never |  | None |
|  |  | Rarely | No |  |
|  |  | Occasionally | No |  |
|  |  | Frequently | Yes |  |
|  |  | Almost constantly | Yes |  |
| **6a** | In the last 7 days, how much did **loss of control of bowel movements** INTERFERE with your usual or daily activities? |  | No | 6a |
| **7*** | In the last 7 days, what was the SEVERITY of your **constipation** at its WORST? | None |  | None |
|  |  | Mild | No |  |
|  |  | Moderate | No |  |
|  |  | Severe | Yes |  |
|  |  | Very severe | Yes |  |
| **8** | In the last 7 days, what was the SEVERITY of your **mouth or throat sores** at their WORST? | None |  | None |
|  |  | Mild | No |  |
|  |  | Moderate | No |  |
|  |  | Severe | Yes |  |
|  |  | Very severe | Yes |  |
| **8a** | In the last 7 days, how much did **mouth or throat sores** INTERFERE with your usual or daily activities? |  | No | 8 |
| **9** | In the last 7 days, what was the SEVERITY of your **watery eyes** (tearing) at their WORST? |  |  | None |
| **9a** | In the last 7 days, how much did **watery eyes** (tearing) INTERFERE with your usual or daily activities? |  | No | 9 |
| **10** | In the last 7 days, what was the SEVERITY of your **dry skin** at its WORST? |  | No | None |
| **11** | In the last 7 days, what was the SEVERITY of your **hand-foot syndrome** (a rash of the hands or feet that can cause cracking, peeling, redness, or pain) at its WORST? | None |  | None |
|  |  | Mild | No |  |
|  |  | Moderate | No |  |
|  |  | Severe | Yes |  |
|  |  | Very severe | Yes |  |
| **12** | In the last 7 days, how OFTEN have you had **flu like symptoms** (hot, cold, shivery and achy)? | Never |  | None |
|  |  | Rarely | No |  |
|  |  | Occasionally | No |  |
|  |  | Frequently | Yes |  |
|  |  | Almost constantly | Yes |  |
| **12a** | In the last 7 days, how much did **flu like symptoms** (hot, cold, shivery and achy) INTERFERE with your usual or daily activities? |  | No | 12 |
| **13** | In the last 7 days, what was the SEVERITY of your **cough** at its WORST? | None |  | None |
|  |  | Mild | No |  |
|  |  | Moderate | No |  |
|  |  | Severe | Yes |  |
|  |  | Very severe | Yes |  |
| **13a** | In the last 7 days, how much did **cough** INTERFERE with your usual or daily activities? |  | No | 13 |
| **14*** | In the last 7 days, what was the SEVERITY of your **shortness of breath** at its WORST? | None |  | None |
|  |  | Mild | No |  |
|  |  | Moderate | No |  |
|  |  | Severe | Yes |  |
|  |  | Very severe | Yes |  |
| **14a** | In the last 7 days, how much did your **shortness of breath** INTERFERE with your usual or daily activities? |  | No | 14 |
| **15*** | In  the last 7 days, what was the SEVERITY of your **fatigue, tiredness, or lack of energy** at its WORST? | None |  | None |
|  |  | Mild | No |  |
|  |  | Moderate | No |  |
|  |  | Severe | Yes |  |
|  |  | Very severe | Yes |  |
| **15a** | In the last 7 days, how much did **fatigue, tiredness, or lack of energy** INTERFERE with your usual or daily activities? |  | No | 15 |
| **16*** | In the last 7 days, what was the SEVERITY of your **problems with** **memory** at their WORST? |  | No | None |
| **16a** | In the last 7 days, how much did **problems with** **memory** INTERFERE with your usual or daily activities? |  | No | 16 |
| **17*** | In the last 7 days, what was the SEVERITY of your **problems with** **concentration** at their WORST? |  | No | None |
| **17a** | In the last 7 days, how much did **problems with concentration** INTERFERE with your usual or daily activities? |  | No | 17 |
| **18*** | In the last 7 days, how OFTEN did you have a **headache**? |  | No | None |
| **18a** | In the last 7 days, what was the SEVERITY of your **headache** at its WORST? | None |  | 18 |
|  |  | Mild | No |  |
|  |  | Moderate | No |  |
|  |  | Severe | Yes |  |
|  |  | Very severe | Yes |  |
| **18b** | In the last 7 days, how much did your **headache** INTERFERE with your usual or daily activities? |  | No | 18 |
| **19** | In the last 7 days, what was the SEVERITY of your **dizziness** at its WORST? | None |  | None |
|  |  | Mild | No |  |
|  |  | Moderate | No |  |
|  |  | Severe | Yes |  |
|  |  | Very severe | Yes |  |
| **19a** | In the last 7 days, how much did **dizziness** INTERFERE with your usual or daily activities? |  | No | 19 |
| **20** | In the last 7 days, what was the SEVERITY of your **blurry vision** at its WORST? |  | No | None |
| **20a** | In the last 7 days, how much did **blurry vision** INTERFERE with your usual or daily activities? |  | No | 20 |
| **21** | In the last 7 days, what was the SEVERITY of **ringing in your ears** at its WORST? | None |  | None |
|  |  | Mild | No |  |
|  |  | Moderate | No |  |
|  |  | Severe | Yes |  |
|  |  | Very severe | Yes |  |
| **22*** | In the last 7 days, what was the SEVERITY of your **numbness or tingling in your hands or feet** at its WORST? | None |  | None |
|  |  | Mild | No |  |
|  |  | Moderate | No |  |
|  |  | Severe | Yes |  |
|  |  | Very severe | Yes |  |
| **22a** | In the last 7 days, how much did **numbness or tingling in your hands or feet** INTERFERE with your usual or daily activities? |  | No | 22 |
| **23** | In the last 7 days, how OFTEN did you have **nosebleeds**? |  | No | None |
| **23a** | In the last 7 days, what was the SEVERITY of your **nosebleeds** at their WORST? | None |  | 23 |
|  |  | Mild | No |  |
|  |  | Moderate | No |  |
|  |  | Severe | Yes |  |
|  |  | Very severe | Yes |  |
| **24*** | In the last 7 days, how OFTEN did you feel **anxiety**? |  | No | None |
| **24a** | In the last 7 days, what was the SEVERITY of your **anxiety** at its WORST? | None |  | 24 |
|  |  | Mild | No |  |
|  |  | Moderate | No |  |
|  |  | Severe | Yes |  |
|  |  | Very severe | Yes |  |
| **24b** | In the last 7 days, how much did **anxiety** INTERFERE with your usual or daily activities? |  | No | 24 |
| **25*** | In the last 7 days, how OFTEN did you have **sad or unhappy feelings**? |  | No | None |
| **25a** | In the last 7 days, what was the SEVERITY of your **sad or unhappy feelings** at their WORST? | None |  | 25 |
|  |  | Mild | No |  |
|  |  | Moderate | No |  |
|  |  | Severe | Yes |  |
|  |  | Very severe | Yes |  |
| **25b** | In the last 7 days, how much did **sad or unhappy feelings** INTERFERE with your usual or daily activities? |  | No | 25 |
| **26*** | In the last 7 days, what was the SEVERITY of your **insomnia** (including difficulty falling asleep, staying asleep, or waking up early) at its WORST? |  | No | None |
| **26a** | In the last 7 days, how much did **insomnia** (including difficulty falling asleep, staying asleep, or waking up early) INTERFERE with your usual or daily activities? |  | No | 26 |
| **27** | In the last 7 days, did you have any **hair loss**? | (INTERFERE options) | No | None |
| **28** | In the last 7 days, did you **lose any fingernails or toenails**? | Yes/No |  | None |
| **29** | In the last 7 days, did you have any **ridges or bumps on your fingernails or toenails**? | Yes/No |  | None |
| **30** | In the last 7 days, did you **bruise easily** (black and blue marks)? | Yes/No |  | None |
| **31** | Do you have any other symptoms that you wish to report? Please list any other symptoms –  **Other Symptom 1:** | Free text box |  | None |
| **31a** | In the last 7 days, what was the SEVERITY of this symptom at its worst? | Mild | No | 31 |
|  |  | Moderate | No |  |
|  |  | Severe | Yes |  |
|  |  | Very severe | Yes |  |
| **32** | **Other Symptom 2:** | Free text box |  | None |
| **32a** | In the last 7 days, what was the SEVERITY of this symptom at its worst? | Mild | No | 32 |
|  |  | Moderate | No |  |
|  |  | Severe | Yes |  |
|  |  | Very severe | Yes |  |
| **33** | **Other Symptom 3:** | Free text box |  | None |
| **33a** | In the last 7 days, what was the SEVERITY of this symptom at its worst? | Mild | No | 33 |
|  |  | Moderate | No |  |
|  |  | Severe | Yes |  |
|  |  | Very severe | Yes |  |
| **34** | **Other Symptom 4:** | Free text box |  | None |
| **34a** | In the last 7 days, what was the SEVERITY of this symptom at its worst? | Mild | No | 34 |
|  |  | Moderate | No |  |
|  |  | Severe | Yes |  |
|  |  | Very severe | Yes |  |
| **35** | **Other Symptom 5:** | Free text box |  | None |
| **35a** | In the last 7 days, what was the SEVERITY of this symptom at its worst? | Mild | No | 35 |
|  |  | Moderate | No |  |
|  |  | Severe | Yes |  |
|  |  | Very severe | Yes |  |

* Symptoms are those identified by Reeve et al. and Trask et al. as being core symptoms for all patients.

^#^ The responses for items are as follows: OFTEN items: Never/Rarely/Occasionally/Frequently/Almost constantly; SEVERITY items: None/Mild/Moderate/Severe/Very Severe; INTERFERENCE items: Not at all/A little bit/Somewhat/Quite a bit/Very much.

**S2. Method of comparison between clinician-recorded AEs in trial/medical records and PRO-AEs**

At the end of the participant’s involvement on study, the research team completed a dedicated CRF relating to clinician-reported toxicity assessments by exploring the patient’s medical and EPCT trial records. This was completed at two timepoints – 1) near to the patient’s first ePRO (online) completion, and 2) near to the patient’s last ePRO completion (note, this could be a week 12 completion, or it could be the last completion prior to the patient being withdrawn).

This aimed to collect any clinician-recorded AEs 7 days either side of the patient’s completion, allowing some flexibility here as the ePRO questionnaire asks about symptoms over the last 7 days, and clinicians often ask patients about symptoms in the last 7 days, but their visit may not fall on the same day as the online completion.

The clinician-recorded data was collected as per CTCAE grades that are used to describe AEs in trial records, ranging from 0-4 (0 = not experienced AE, and a higher number indicates more severe AE), but it is worth noting that not all symptoms have a 3-4 CTCAE category. There was also an ungraded category if an AE was mentioned but not graded, and a category if the AE was not mentioned at all in the trial/medical records (i.e. we did not assume that no mention equalled not having the AE).

The research team had more access to participant medical records at hospital 1, being physically based at that location. It is also notable that each EPCT differed in the AE logs it kept, with some trials keeping detailed visit-specific AE log tables, whereas others provided records on an overall AE sheet recording the type of AE, its grade and its start and stop dates. Furthermore, some trials had pre-set AEs of interest that they recorded against each time the patient was reviewed.

This data was explored in two ways:

1. *Comparative presence/absence of symptoms*

All symptoms (patient and clinician) were recoded into whether they had experienced the symptom or not (yes/no). Symptoms were then compared to see if patients and clinicians *agreed* on whether the symptom had been experienced or not (agreed = both indicating ‘yes’ or both indicating ‘no’), patient-reported only, or clinician-reported only. This was done on each core/initial symptom (e.g. frequency of pain), rather than on sub-questions which were only answered if a patient had experienced the symptom. If a symptom was not mentioned or ungraded within the clinical/trial records (clinician scores), comparison to the patient data was not possible.

1. *Exploration of ‘not mentioned’ data*

Where ePRO symptoms were considered not mentioned in medical/trial records within the above point, the ePRO data was explored to illustrate whether any symptoms were missed. This is only presented relating to the symptoms that were present (score 1-4) on ePRO reports.

**S3 Early phase trial participation amongst the ePRIME participants (n=23)**

**S4. Symptom level comparison between patient and clinician records at baseline and last completion timepoint**

*Table 4.1 Presence/absence symptom agreement between patient***^#^** *and clinician at baseline timepoint (n=23)*

| **Symptom** | **Agreement** | | **Patient reported only** | | **Clinician reported only** | | **Not mentioned** | | **Ungraded** | |
| --- | --- | --- | --- | --- | --- | --- | --- | --- | --- | --- |
|  | n | % | n | % | n | % | n | % | n | % |
| Fatigue (S) | 14 | 60.9 | 1 | 4.3 | 1 | 4.3 | 7 | 30.4 | 0 | 0 |
| Pain (F) | 10 | 43.5 | 2 | 8.7 | 1 | 4.3 | 9 | 39.1 | 1 | 4.3 |
| Diarrhoea (F) | 10 | 43.5 | 2 | 8.7 | 0 | 0 | 11 | 47.8 | 0 | 0 |
| Lose control bowels* (F) | 9 | 39.1 | 0 | 0 | 3 | 13.0 | 11 | 47.8 | 0 | 0 |
| Vomiting (F) | 9 | 39.1 | 0 | 0 | 0 | 0 | 14 | 60.9 | 0 | 0 |
| Nausea (F) | 8 | 34.8 | 3 | 13.0 | 0 | 0 | 12 | 52.2 | 0 | 0 |
| Mucositis (mouth and throat sores) (S) | 8 | 34.8 | 1 | 4.3 | 1 | 4.3 | 13 | 65.5 | 0 | 0 |
| Alopecia (hair loss)^$^ (F) | 7 | 30.4 | 0 | 0 | 0 | 0 | 14 | 60.9 | 1 | 4.3 |
| Constipation (S) | 6 | 26.1 | 2 | 8.7 | 0 | 0 | 15 | 65.2 | 0 | 0 |
| Hand-foot syndrome (S) | 6 | 26.1 | 0 | 0 | 2 | 8.7 | 15 | 65.2 | 0 | 0 |
| Peripheral sensory neuropathy (S) | 5 | 21.7 | 1 | 4.3 | 1 | 4.3 | 16 | 69.6 | 0 | 0 |
| Decreased appetite (S) | 3 | 13.0 | 2 | 8.7 | 0 | 0 | 18 | 78.3 | 0 | 0 |
| Dyspnea (shortness of breath) (S) | 3 | 13.0 | 1 | 4.3 | 0 | 0 | 19 | 82.6 | 0 | 0 |
| Cough (S) | 3 | 13.0 | 0 | 0 | 0 | 0 | 20 | 87.0 | 0 | 0 |
| Tinnitus (ringing in ears) (S) | 2 | 8.7 | 0 | 0 | 0 | 0 | 21 | 91.3 | 0 | 0 |
| Bruising^$^ (Y/N) | 2 | 8.7 | 0 | 0 | 1 | 4.3 | 19 | 82.6 | 0 | 0 |
| Epistaxis (nosebleeds) (F) | 2 | 8.7 | 0 | 0 | 0 | 0 | 21 | 91.3 | 0 | 0 |
| Dry skin (S) | 2 | 8.7 | 0 | 0 | 1 | 4.3 | 20 | 87.0 | 0 | 0 |
| Anxiety (F) | 1 | 4.3 | 1 | 4.3 | 1 | 4.3 | 20 | 87.0 | 0 | 0 |
| Depression (F) | 1 | 4.3 | 1 | 4.3 | 0 | 0 | 21 | 91.3 | 0 | 0 |
| Dizziness (S) | 1 | 4.3 | 0 | 0 | 0 | 0 | 22 | 95.7 | 0 | 0 |
| Headache (F) | 1 | 4.3 | 0 | 0 | 0 | 0 | 22 | 95.7 | 0 | 0 |
| Insomnia^$^ (S) | 0 | 0 | 0 | 0 | 0 | 0 | 22 | 95.7 | 0 | 0 |
| Concentration impairment (S) | 0 | 0 | 0 | 0 | 0 | 0 | 23 | 100 | 0 | 0 |
| Memory impairment (S) | 0 | 0 | 0 | 0 | 0 | 0 | 23 | 100 | 0 | 0 |
| Blurred vision (S) | 0 | 0 | 0 | 0 | 0 | 0 | 23 | 100 | 0 | 0 |
| Watering eyes (S) | 0 | 0 | 0 | 0 | 0 | 0 | 23 | 100 | 0 | 0 |
| **Total counts across symptoms** | **113** | **18.2%** | **17** | **2.7%** | **12** | **1.9%** | **474** | **76.5%** | **2** | **0.3%** |

^$ One missing response for these symptoms as patient stopped completing questionnaire part way through^

^# This comparison is only done on each of the initial ePRO items, rather than items that only appeared if the patient indicated experiencing the symptom^

^F = Frequency item, S = Severity item, Y/N = yes/no item^

^* The lost of bowel control PRO item is compared to the diarrhoea clinician rating^

*Table 4.2 Presence/absence symptom agreement between patient^#^ and clinician at last completion timepoint (n=22)*

| **Symptom** | **Agreement** | | **Patient reported only** | | **Clinician reported only** | | **Not mentioned** | | **Ungraded** | |
| --- | --- | --- | --- | --- | --- | --- | --- | --- | --- | --- |
|  | n | % | n | % | n | % | n | % | n | % |
| Fatigue (S) | 15 | 68.2 | 2 | 9.1 | 1 | 4.5 | 4 | 18.2 | 0 | 0 |
| Diarrhoea (F) | 10 | 45.5 | 3 | 13.6 | 0 | 0 | 8 | 36.4 | 1 | 4.5 |
| Lose control bowels* (F) | 9 | 40.9 | 1 | 4.5 | 3 | 13.6 | 8 | 36.4 | 1 | 4.5 |
| Nausea (F) | 8 | 36.4 | 1 | 4.5 | 0 | 0 | 13 | 59.1 | 0 | 0 |
| Vomiting (F) | 7 | 31.8 | 1 | 4.5 | 1 | 4.5 | 13 | 59.1 | 0 | 0 |
| Mouth and throat sores (S) | 7 | 31.8 | 1 | 4.5 | 0 | 0 | 14 | 63.6 | 0 | 0 |
| Pain (F) | 6 | 27.3 | 2 | 9.1 | 3 | 13.6 | 9 | 40.9 | 2 | 9.1 |
| Alopecia (F) | 6 | 27.3 | 0 | 0 | 0 | 0 | 16 | 72.7 | 0 | 0 |
| Constipation (S) | 5 | 22.7 | 3 | 13.6 | 0 | 0 | 14 | 63.6 | 0 | 0 |
| Peripheral sensory neuropathy (S) | 5 | 22.7 | 3 | 13.6 | 1 | 4.5 | 13 | 59.1 | 0 | 0 |
| Dyspnea (S) | 5 | 22.7 | 1 | 4.5 | 0 | 0 | 16 | 72.7 | 0 | 0 |
| Hand-foot syndrome (S) | 5 | 22.7 | 3 | 13.6 | 0 | 0 | 14 | 63.6 | 0 | 0 |
| Decreased appetite (S) | 4 | 18.2 | 1 | 4.5 | 0 | 0 | 17 | 77.3 | 0 | 0 |
| Bruising (Y/N) | 3 | 13.6 | 0 | 0 | 0 | 0 | 19 | 86.4 | 0 | 0 |
| Cough (S) | 2 | 9.1 | 0 | 0 | 2 | 9.1 | 18 | 81.8 | 0 | 0 |
| Epistaxis (F) | 2 | 9.1 | 0 | 0 | 0 | 0 | 20 | 90.9 | 0 | 0 |
| Tinnitus (S) | 2 | 9.1 | 0 | 0 | 0 | 0 | 20 | 90.9 | 0 | 0 |
| Anxiety (F) | 1 | 4.5 | 0 | 0 | 1 | 4.5 | 19 | 86.4 | 1 | 4.5 |
| Depression (F) | 1 | 4.5 | 0 | 0 | 0 | 0 | 21 | 95.5 | 0 | 0 |
| Concentration impairment (S) | 1 | 4.5 | 0 | 0 | 0 | 0 | 21 | 95.5 | 0 | 0 |
| Dry skin (S) | 1 | 4.5 | 1 | 4.5 | 0 | 0 | 20 | 90.9 | 0 | 0 |
| Blurred vision (S) | 1 | 4.5 | 0 | 0 | 0 | 0 | 20 | 90.9 | 1 | 4.5 |
| Watering eyes (S) | 1 | 4.5 | 0 | 0 | 0 | 0 | 21 | 95.5 | 0 | 0 |
| Memory impairment (S) | 0 | 0 | 0 | 0 | 0 | 0 | 22 | 100 | 0 | 0 |
| Insomnia (S) | 0 | 0 | 0 | 0 | 0 | 0 | 22 | 100 | 0 | 0 |
| Dizziness (S) | 0 | 0 | 0 | 0 | 0 | 0 | 22 | 100 | 0 | 0 |
| Headaches (F) | 0 | 0 | 0 | 0 | 0 | 0 | 21 | 95.5 | 1 | 4.5 |
| **Total counts across symptoms** | **107** | **18.0%** | **23** | **3.9%** | **12** | **2.0** | **445** | **74.9%** | **7** | **1.2%** |

^# This comparison is only done on each of the initial ePRO items, rather than items that only appeared if the patient indicated experiencing the symptom^

^F = Frequency item, S = Severity item, Y/N = yes/no item^

^* The lost of bowel control PRO item is compared to the diarrhoea clinician rating^

**S5. Exploration of ‘not mentioned’ clinician symptoms and ePRO scoring at baseline and last completion timepoint**

*Table 5.1 Comparison between clinician ‘not mentioned’ symptoms and ePRO scoring (0-4) at baseline completion (n=23)*

| **Symptom** | **Total Not Mentioned by clinician*** | **Patient scored 1 (Rarely/Mild)** | | **Patient scored 2 (Occasionally/ Moderate)** | | **Patient scored 3 (Frequently/ Severe)** | | **Patient scored 4 (Almost Constantly/ Very Severe)** | |
| --- | --- | --- | --- | --- | --- | --- | --- | --- | --- |
|  | n | n | % | N | % | n | % | n | % |
| Depression | 13 | 6 | 46.2 | 6 | 46.2 | 1 | 7.7 | 0 | 0 |
| Insomnia | 12 | 6 | 50.0 | 6 | 50.0 | 0 | 0 | 0 | 0 |
| Anxiety | 9 | 4 | 44.4 | 3 | 33.3 | 1 | 11.1 | 1 | 11.1 |
| Decreased appetite | 9 | 5 | 55.6 | 3 | 33.3 | 1 | 11.1 | 0 | 0 |
| Dizziness | 7 | 4 | 57.1 | 3 | 42.9 | 0 | 0 | 0 | 0 |
| Pain | 5 | 3 | 60.0 | 2 | 40.0 | 0 | 0 | 0 | 0 |
| Fatigue | 5 | 2 | 40.0 | 2 | 40.0 | 1 | 20.0 | 0 | 0 |
| Concentration issues | 5 | 3 | 60.0 | 2 | 40.0 | 0 | 0 | 0 | 0 |
| Memory issues | 5 | 5 | 100.0 | 0 | 0.0 | 0 | 0 | 0 | 0 |
| Peripheral neuropathy | 5 | 5 | 100.0 | 0 | 0.0 | 0 | 0 | 0 | 0 |
| Headache | 5 | 1 | 20.0 | 3 | 60.0 | 0 | 0 | 1 | 20.0 |
| Dry skin | 5 | 4 | 80.0 | 1 | 20.0 | 0 | 0 | 0 | 0 |
| Dyspnea | 5 | 3 | 60.0 | 1 | 20.0 | 1 | 20.0 | 0 | 0 |
| Cough | 5 | 3 | 60.0 | 2 | 40.0 | 0 | 0 | 0 | 0 |
| Constipation | 4 | 3 | 75.0 | 1 | 25.0 | 0 | 0 | 0 | 0 |
| Diarrhoea | 4 | 3 | 75.0 | 1 | 25.0 | 0 | 0 | 0 | 0 |
| Blurry vision | 4 | 4 | 100.0 | 0 | 0.0 | 0 | 0 | 0 | 0 |
| Tinnitus | 4 | 2 | 50.0 | 2 | 50.0 | 0 | 0 | 0 | 0 |
| Nausea | 3 | 2 | 66.7 | 1 | 33.3 | 0 | 0 | 0 | 0 |
| Watery eyes | 3 | 3 | 100.0 | 0 | 0.0 | 0 | 0 | 0 | 0 |
| Alopecia | 3 | 3 | 100.0 | 0 | 0.0 | 0 | 0 | 0 | 0 |
| Vomiting | 2 | 2 | 100.0 | 0 | 0.0 | 0 | 0 | 0 | 0 |
| Bruise | 1 | 1 | 100.0 | 0 | 0.0 | 0 | 0 | 0 | 0 |
| Nosebleeds | 1 | 1 | 100.0 | 0 | 0.0 | 0 | 0 | 0 | 0 |
| Hand and foot syndrome | 1 | 1 | 100.0 | 0 | 0.0 | 0 | 0 | 0 | 0 |
| Lose control bowel | 1 | 0 | 0.0 | 1 | 100.0 | 0 | 0 | 0 | 0 |
| Mucositis | 0 | 0 | 0.0 | 0 | 0.0 | 0 | 0 | 0 | 0 |
| **Total** | **126** | **79** | **62.7** | **40** | **31.7** | **5** | **4.0** | **2** | **1.6** |

^* Excluding N=348 score 0 (not experienced) ePRO responses^

*Table 5.2 Comparison between clinician ‘not mentioned’ symptoms and ePRO scores (0-4) at last completion (n=22)*

| **Symptom** | **Total Not Mentioned by clinician*** | **Patient scored 1 (Rarely/Mild)** | | **Patient scored 2 (Occasionally/ Moderate)** | | **Patient scored 3 (Frequently/ Severe)** | | **Patient scored 4 (Almost Constantly/Very Severe)** | |
| --- | --- | --- | --- | --- | --- | --- | --- | --- | --- |
|  | n | n | % | n | % | n | % | n | % |
| Insomnia | 12 | 7 | 58.3 | 5 | 41.7 | 0 | 0 | 0 | 0 |
| Depression | 11 | 6 | 54.5 | 4 | 36.4 | 0 | 0 | 1 | 9.1 |
| Cough | 9 | 9 | 100.0 | 0 | 0.0 | 0 | 0 | 0 | 0 |
| Anxiety | 8 | 4 | 50.0 | 4 | 50.0 | 0 | 0 | 0 | 0 |
| Dry skin | 8 | 5 | 62.5 | 3 | 37.5 | 0 | 0 | 0 | 0 |
| Decreased appetite | 6 | 5 | 83.3 | 1 | 16.7 | 0 | 0 | 0 | 0 |
| Dyspnea | 6 | 5 | 83.3 | 1 | 16.7 | 0 | 0 | 0 | 0 |
| Headache | 5 | 2 | 40.0 | 3 | 60.0 | 0 | 0 | 0 | 0 |
| Alopecia | 4 | 2 | 50.0 | 2 | 50.0 | 0 | 0 | 0 | 0 |
| Constipation | 4 | 2 | 50.0 | 2 | 50.0 | 0 | 0 | 0 | 0 |
| Fatigue | 4 | 2 | 50.0 | 1 | 25.0 | 1 | 25.0 | 0 | 0 |
| Concentration issues | 4 | 3 | 75.0 | 1 | 25.0 | 0 | 0 | 0 | 0 |
| Watery eyes | 4 | 4 | 100.0 | 0 | 0.0 | 0 | 0 | 0 | 0 |
| Memory issues | 3 | 2 | 66.7 | 1 | 33.3 | 0 | 0 | 0 | 0 |
| Tinnitus | 3 | 2 | 66.7 | 1 | 33.3 | 0 | 0 | 0 | 0 |
| Peripheral neuropathy | 2 | 2 | 100.0 | 0 | 0.0 | 0 | 0 | 0 | 0 |
| Dizziness | 2 | 2 | 100.0 | 0 | 0.0 | 0 | 0 | 0 | 0 |
| Pain | 2 | 2 | 100.0 | 0 | 0.0 | 0 | 0 | 0 | 0 |
| Bruise | 2 | 2 | 100.0 | 0 | 0.0 | 0 | 0 | 0 | 0 |
| Diarrhoea | 2 | 1 | 50.0 | 1 | 50.0 | 0 | 0 | 0 | 0 |
| Nausea | 1 | 1 | 100.0 | 0 | 0.0 | 0 | 0 | 0 | 0 |
| Nosebleeds | 1 | 1 | 100.0 | 0 | 0.0 | 0 | 0 | 0 | 0 |
| Blurry vision | 1 | 1 | 100.0 | 0 | 0.0 | 0 | 0 | 0 | 0 |
| Vomiting | 0 | 0 | 0.0 | 0 | 0.0 | 0 | 0 | 0 | 0 |
| Mucositis | 0 | 0 | 0.0 | 0 | 0.0 | 0 | 0 | 0 | 0 |
| Hand and foot syndrome | 0 | 0 | 0.0 | 0 | 0.0 | 0 | 0 | 0 | 0 |
| Lose control bowel | 0 | 0 | 0.0 | 0 | 0.0 | 0 | 0 | 0 | 0 |
| **Total** | **104** | **72** | **69.2** | **30** | **28.8** | **1** | **0.96** | **1** | **0.96** |

^* Excluding N=341 score 0 (not experienced) ePRO responses^

**S6. Counts of symptoms generating notification email alerts**

*Table 6.1 Type of symptoms generating notifications*

| **Individual symptom alert** | **Overall Count** | **Count of severe (scored 3)** | **Count of very severe (scored 4)** |
| --- | --- | --- | --- |
| Fatigue | 17 | 17 | 0 |
| Other symptoms (see Table 4.2 below) | 14 | 11 | 3 |
| Pain | 12 | 11 | 1 |
| Lack of Appetite | 6 | 6 | 0 |
| Diarrhoea | 6 | 6 | 0 |
| Cough | 5 | 5 | 0 |
| Hand-foot syndrome | 5 | 5 | 0 |
| Anxiety | 3 | 3 | 0 |
| Flu-like symptoms | 3 | 3 | 0 |
| Dyspnea (shortness of breath) | 2 | 2 | 0 |
| Headache | 2 | 2 | 0 |
| Constipation | 2 | 2 | 0 |
| Nausea | 1 | 1 | 0 |
| Depression | 1 | 1 | 0 |
| Mucositis (mouth sores) | 1 | 1 | 0 |
| Vomiting | 1 | 1 | 0 |

*Table 6.2 Free-text ‘other’ symptoms reported by patients generating notifications*

| **Other symptoms generating notifications** | **Count** | **Number of participants** |
| --- | --- | --- |
| Water infection/pain when passsing urine | 4 | 2 |
| Pain in knee | 2 | 1 |
| Blood in urine | 1 | 1 |
| Rash on feet and legs | 1 | 1 |
| High temperature | 1 | 1 |
| Back pains | 1 | 1 |
| Achy muscles everywhere | 1 | 1 |
| Sore feet/blistering through gardening | 1 | 1 |
| ‘Not a good week’ | 1 | 1 |
| ‘Due to hospital I am late my trial’ | 1 | 1 |

**S7. Summary of patient feedback and other issues recording during the study**

*Table 7.1-Summary of patient survey and interview feedback (N=21)*

| **Areas explored** | Survey (N=4) | Interviews (N=17) |
| --- | --- | --- |
| Learning to use system | 4/4 Very Easy | Some mentioned not being IT savvy but generally positive comments *“I mean it’s a lot of years since I used a computer so for me it must have been easy, it was really simple.”* |
| Accessibility | 4/4 Very Easy | 3 had some difficulties accessing the system (for 2 these were resolved; 1 gave up). |
| Answering questions | 2 Easy; 1 Very Easy; 1 Neither Easy nor Difficult | Most participants found it easy to answer the questions. One indicated some difficulties teasing out cancer/treatment symptoms or just general tiredness *“Well something like is it says do you get tired, well was that like before or just, but not really because these things happen because I tried to keep more active as well which probably makes more, more tired as well - I mean cementing every day and mixing cement and things like that but I think the question is easy to answer”* |
| Time taken to complete | 4/4 About right | Most were happy with time taken – 5-10 minutes. One mentioned repetitiveness and one indicated the layout of questions on pages could have helped completion time *“I mean to me it would make sense to have more questions on each page you know rather than having to click each time, it felt quite clunky from that sort of design perspective.”* |
| Relevance of symptoms | 2 Quite relevant; 1 Very relevant; 1 Very few relevant | Most found the selection of symptoms relevant or could recognise importance of them even if not experienced. Some commented that they didn’t tick yes to many but that *“from speaking to the nurses they are known side effects so yeah I felt quite lucky really that I was only having to fill in.”* but another commented *“if you’ve got nothing to report, why do I have to do it?”* |
| Frequency (weekly) and timescale (12 weeks) | 3/4 happy to complete every week; 1 unsure | Most were happy with weekly completions (a couple would have preferred every 2 weeks). A number indicated they’d be happy to continue longer than 12 weeks, but not all. One participant said: *"I think it should be used all the time, I think it should just be a standard part of clinical trial practice for all patients on all trials."* |
| Contact with trial team about information | 2 Yes; 2 No | Three indicated they were contacted by their trial team after reporting a symptom on the system. Six stated they had no contact with trial team about information they reported via system. |
| Positives | Hoping to help future patients. Awareness of symptoms. | Free-text symptom box.  Reminders helpful.  Prompts patient to consider (and monitor) possible symptoms, and reassures you if patient not getting symptoms. *“I think it’s a good thing for all clinical trials… I think ePRIME probably gets patients to report things that they might not have done."*  Easier to complete than trial AE log (paper, open ended). *“I found the online one a lot easier than, than doing it, because they give you a booklet where you can like put in the dates when you’re not feeling very well and all the things like that, but I found the online one a lot easier to fill in.”* |
| Negatives | If didn’t complete on time, it could get confusing.  Filling in for patient unable to use computer. | 8 said none.  IT issues (3 participants mentioned)  Already being seen regular, takes up time and prefers direct personal contact.  A chore to report after already relaying to doctor.  System layout for other questions was confusing.  Technical issue regarding amending a symptom.  Mental state at the time made it very challenging (note, patient withdrew in week 1).  Causing panic amongst research nurse and risk of being taken off trial, which cause participant a lot of stress (note, patient withdrew in week 5). |
| Suggestions for improvements | A column for how coped with symptoms to help other patients. | Free-text box at the end/to explain (3 participants).  Another reminder/window of opportunity for completing.  Option of completing/being reminded to complete more than once a week.  Knowing it was going to clinicians/trial team.  More questions on each page – it was clunky (2 participants).  Layout for other questions – narrow bar.  Function to stop you completing more than once.  4 stated no improvements needed. |

*Table 7.2 Issues recorded by the research team during the study*

| **Type of issue** | **Number logged** |
| --- | --- |
| **System level issues** | **18** |
| QTool access issue – reported by patients | **3** |
| Reminder issues:   - *Email failed to send due to tickbox about preference not being selected in database* - *SMS reminder issue* - *Researcher put wrong email address in database* | **3**  *1*  *1*  *1* |
| Initial set up issues:  *- QTool - questionnaire not made live*  *- no access to live database (MACRO)* | **2**  *1*  *1* |
| Registration system – couldn’t hear username/password so patient didn’t complete baseline | **2** |
| System outages:  *- major issue (5 weeks) - reported on 4 occasions by patients trying to access*  *- 2 day issue* | **2**  *1*  *1* |
| System alert issue:   - *alert received but when checked no high symptoms (patient may have changed but this isn’t recorded by system)* - *alert not received for severe symptoms (possibly due to university email server issue)* | **5**  *1*  *4* |
| QTool system error during baseline completion (maybe linked to university email server issue above) | **1** |
| **Patient level issues** | **4** |
| Access - lost username | 1 |
| Patient concerned about all data going to research nurses (worried will be taken off EPCT) - decided to withdraw. | 1 |
| Patient confused about reminder timing | 1 |
| Patient unsure if still to complete as chemo stopped | 1 |
| **Study level issues** | **4** |
| Non-completion of baseline QTool:   - *Researcher took wrong link for baseline questionnaire* - *Research nurse thought needed PI signature first* - *Patient needed reminder after few days (2 participants)* | *1*  *1*  *2* |
| **Total** | **26** |

QTool = the online patient-reported system
